# Nanoparticles of Metal-Organic Cages Overcoming Drug Resistance in Ovarian Cancer

**DOI:** 10.3389/fchem.2019.00039

**Published:** 2019-02-01

**Authors:** Han Wang, Zihan Qiu, He Liu, Amarasooriya M. D. S. Jayawardhana, Zhizhou Yue, Hala Daghlas, David J. Bowers, Bansidhar Datta, Yao-Rong Zheng

**Affiliations:** ^1^Department of Chemistry and Biochemistry, Integrated Sciences Building, Kent State University, Kent, OH, United States; ^2^Science Research Laboratory, Department of Chemistry and Biochemistry, Kent State University, Kent, OH, United States

**Keywords:** metal-organic cages, nanoprecipitation, DNA damage, host-guest chemistry, drug resistance

## Abstract

A long-standing challenge in the treatment of ovarian cancer is drug resistance to standard platinum-based chemotherapy. Recently, increasing attention has been drawn to the use of self-assembled metal-organic complexes as novel therapeutics for cancer treatment. However, high hydrophobicity that is often associated with these structures lowers their solubility and hinders their clinical translation. In this article, we present a proof-of-concept study of using nanoprecipitation to formulate the hydrophobic metal-organic cages and facilitate their use in treating chemoresistant ovarian cancer. The Pt_6_L_4_ Cage **1** is an octahedral cage formed by self-assembly of six 1,10-phenanthroline-Pt(II) centers and four 2,4,6-tris(4-pyridyl)-1,3,5-triazine ligands (L). Cage **1** is able to trigger DNA damage and exhibits promising *in vitro* potency against a panel of human ovarian cancer cell lines. However, due to the large portion of aromatic components, this cage structure has very limited solubility in cell culture media (<20μM). Notably, upon nanoformulation by using fluorescein (**2**) and a pegylated anionic polymer (**3**), the concentration of Cage **1** can reach up to 0.4 mM. Production of the nanoparticles of metal-organic cages (nMOC) is driven by the formation of the 1:1 host-guest complex of **1** and **2** in aqueous solution, which then form nanoprecipitation in presence of poly glutamic acid-*b*-poly ethylene glycol (**3**). The resulted nMOC are about 100 nm in diameter, and they serve as a delivery platform that slowly releases the therapeutic content. The use of fluorescein facilitates monitoring cell entry of nMOC and drug release using flow cytometry. Finally, comparing to cisplatin, the nMOC exhibit comparable *in vitro* efficacy against a panel of human cancer cell lines, and notably, it shows a much lower resistance factor against chemoresistant ovarian cancer cell lines.

## Introduction

Ovarian cancer is the most lethal gynecologic cancer and ranks as the fifth leading cause of death for women in the US. Each year, about 250,000 women are diagnosed with ovarian cancer in the US, and approximately half of these patients die within 5 years after their initial diagnosis (Siegel et al., 2015). The low survival rate of ovarian cancer is mainly attributed to the development of drug resistance to the standard platinum chemotherapy (Holmes, 2015). The FDA-approved platinum drugs, cisplatin and carboplatin, are widely used as the first line treatment for ovarian cancer patients in the US and worldwide (Kelland, 2007). Initially, the patients show good responses to these compounds, but after about 12–24 months, patients commonly develop cancer relapse with drug resistance. Currently, there are very few alternative treatment options after the development of drug resistance to these platinum compounds. Thus, there is an urgent need for new, alternative therapeutics to combat chemoresistant ovarian cancer. In the past, the search of new drug candidates has largely focused on mononuclear metal complexes similar to structure of cisplatin, but there are rarely any clinically relevant breakthroughs (Wilson and Lippard, 2014; Zheng et al., 2014). Macromolecular polynuclear metal complexes emerges as a new type of therapeutic candidates to widen the spectrum of activity of current FDA-approved platinum drugs, however, studies of the therapeutic effects of such compounds are still limited (Farrell, 2015).

Metallosupramolecular coordination complexes represent a novel type of metal-organic complexes constructed by coordination-driven self-assembly (Cook and Stang, 2015). They are ordered, discrete macromolecular structures obtained by controlled aggregation of metal centers and organic ligands, and these ensembles exhibit well-defined sizes and shapes, such as metal-organic polyhedra, polygons, cages, and spheres, etc., (Harris et al., 2013; Xu et al., 2015). Due to the unique structural features, self-assembled metal-organic complexes have led to a large variety of novel research in chemistry, material science, and biology. In recent years, studies about applying such metal-organic complexes in cancer research has attracted increasing attention (Hannon, 2007; Therrien, 2012; Cook et al., 2013; Kaiser et al., 2016; Samanta et al., 2017). Stang, Chi, Therrien, Crowley, and Zheng have respectively published their works on designing new metal-organic polyhedra and cages and testing their anti-cancer efficacy (Lewis et al., 2012; Vajpayee et al., 2013; Grishagin et al., 2014; Zheng et al., 2015; Yue et al., 2018b). However, high hydrophobicity that is often associated with these structures lowers their solubility and hinders their clinical translation (Cook et al., 2013; Cook and Stang, 2015). Therefore, additional formulation of metallosupramolecular coordination complexes is critically important to resolve this issue in order to further advance the field toward clinical translation.

**Scheme 1:**
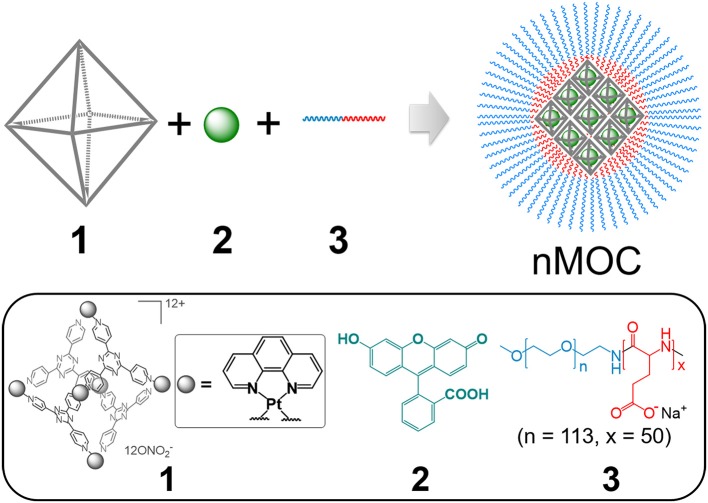
Graphical representation of the formation of nanoparticles of metal-organic cages (nMOC): Cage 1 and fluorescein (2) form a host-guest complex in a 1:1 ratio in aqueous solution, and the use of MPEG_5k_-PGA_50_ anionic block copolymer (3) enables the nanoprecipitation of this complex into nMOC, therefore promoting solubility of this cytotoxic hydrophobic cage.

**Figure 1:**
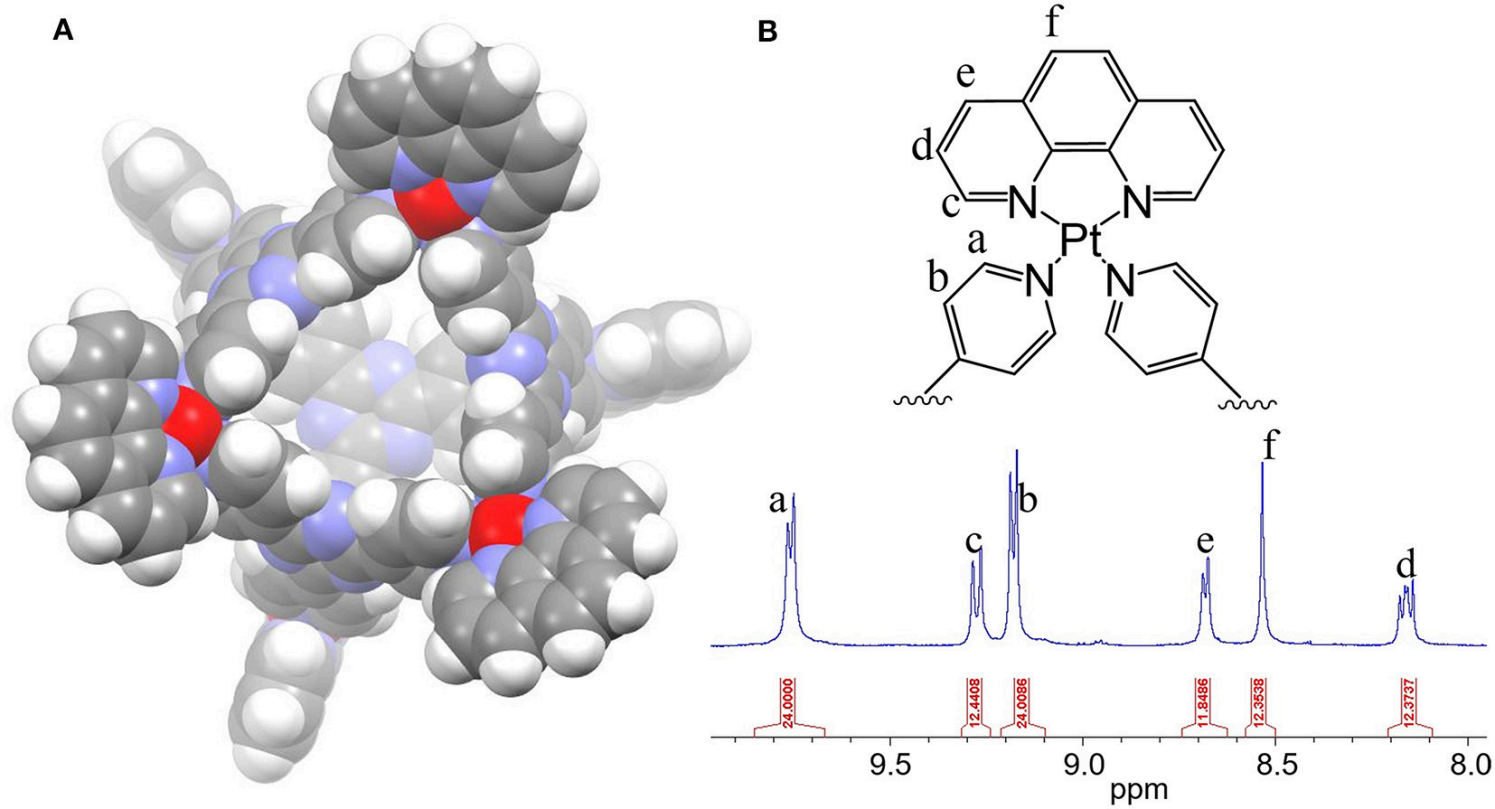
**(A)** The 3D model of the Pt_6_L_4_ metal-organic cage (1) and **(B)** its ^1^H NMR spectrum in DMSO-d_6_.

In this article, we present a proof-of-concept study of using nanoprecipitation to formulate the hydrophobic metal-organic cages and facilitate their use in treating chemoresistant ovarian cancer. As shown in Scheme 1, we can formulate Cage **1**, a cytotoxic hydrophobic metal-organic cage (Figure 1A) with low solubility, using fluorescein (**2**) and a pegylated anionic polymer (**3**). Driven by the host-guest interactions and electrostatic interactions, combination of the three components produces nanoparticles of metal-organic cages (nMOC) covered by PEG, therefore promoting its solubility. The resulted nMOC shows promising anticancer activities against chemoresistant ovarian cancer cells.

## Materials and Methods

### General Information

All reagents were purchased from Strem, Aldrich or Alfa and used without further purification. Methoxy polyethylene glycol-*block*-polyglutamic acid (MPEG_5k_-PGA_50_) (**3**) was purchased from Polypeptide Therapeutic Solutions SL (Valencia, Spain). All reactions were carried out under normal atmospheric conditions. Deuterated solvents were purchased from Cambridge Isotope Laboratory (Andover, MA). The ^1^H NMR spectra were recorded on a Bruker AMX 400 NMR in the Department of Chemistry and Biochemistry at Kent State University. Chemical shifts in ^1^H NMR spectra were internally referenced to solvent signals (DMSO at δ = 2.50 ppm). Elemental analysis was performed on a TruSpec Micro, CHNS analyzer from LECO Corporation. UV-Vis spectra were recorded on a VWR UV-1600PC scanning spectrophotometer. Fluorescence spectra were obtained on a Cary Eclipse fluorescence spectrophotometer. Graphite furnace atomic absorption spectroscopic (GFAAS) measurements were taken on a PerkinElmer PinAAcle 900Z spectrometer. Fluorescence images were acquired using an Olympus IX70 inverted epifluorescence microscope equipped with a digital CCD camera (QImaging). Images were processed and intensities were quantified with ImageJ software (NIH). Dynamic light scattering and zeta-potential analysis were carried out using a Horiba SZ-100 particle analyzer. 3D computational modeling was performed using Schrödinger Macromodel Suite based on MMFF force field. Flow cytometry was carried out on a BD Bioscience Accuri C6 flow cytometer. Cryo-TEM specimens were prepared using thin-film plunge freezing in a FEI Vitrobot at Liquid Crystal Institute at Kent State University. The vitrified specimens were mounted onto a Gatan 626.DH cryo-holder and transferred into a FEI Tecnai F20 TEM. Cryo-TEM observation was performed using low-dose mode. The detailed experimental setup and procedure are reported previously (Gao et al., 2014).

### Synthesis of the Metal-Organic Cage (1)

Synthesis of the Pt_6_L_4_ cage was modified from the previous reports (Zheng et al., 2016; Yue et al., 2018a). Pt(phen)(NO_3_)_2_ (15.0 mg, 30 μmol) and 2,4,6-Tri(4-pyridyl)-1,3,5-triazine (6.0 mg, 19 μmol) were mixed in a microwave reaction vial (2–5 mL), followed by the addition of 4 mL H_2_O and 4 μL of 65% HNO_3_. The reaction vial was then sealed and placed in a Biotage Initiator Microwave Synthesizer. The mixture was stirred at 900 rpm and heated using a multi-step sequence (a. 90°C, 100 W for 30 min; b. 150°C, 150 W for 60 min). The mixture was centrifuged to remove possible precipitations. Then, the supernatant was collected, and the solvent was removed under reduced pressure. The resulted white solid was washed with 1 mL of water under sonication. After removing water by centrifugation, the final product was dried in vacuum. Yield: 15.7 mg, 78%. ^1^H NMR (400 MHz, DMSO-d_6_) δ 9.75 (d, *J* = 6.4 Hz, 24H, α-Pyridine), 9.26 (d, *J* = 8.0 Hz, 12H, phen), 9.16 (d, *J* = 6.8 Hz, 24H, β-Pyridine), 8.67 (d, *J* = 4.2 Hz, 12H, phen), 8.52 (d, *J* = 2.8 Hz, 12H, phen), 8.14 (m, 12H, phen). Anal. Calcd for C_144_H_96_N_48_O_36_Pt_6_·(H_2_O)_6_: C, 39.73; H, 2.50; N, 15.44. Found: C, 39.56; H, 2.25; N, 15.09.

### Preparation of the Nanoparticles of Metal-Organic Cages (nMOC)

To a volume of 1 mL of PBS solution of MPEG_5k_-PGA_50_ (**3**) (1.00 mg/mL) and fluorescein (**2**) (30.0 μL, 13.0 mM) was added the DMSO solution containing the metal-organic cage (**1**) (90.0 μL, 2.00 mM) under shaking at 900 rpm at room temperature (R.T.). The mixture was shaken for 10 min further at R.T. Significant color change from yellow green to red could be observed during the process, which is associated with the formation of the host-guest complex. The resulted red solution was filtered using a 0.2 μm PTFE syringe filter. The filtrate was further purified using centrifugal filtration (MWCO = 30 kDa). The solution of the nanoparticles was concentrated to a volume of 0.8 mL. The Pt content was determined to be 768 μM using GFAAS. Each cage molecule contains six Pt centers. Therefore, the concentration of **1** in nMOC is 128 μM in PBS. Yield: 56.7%. This sample can be further concentrated up to 0.4 mM in PBS by centrifugal filtration (MWCO = 30 kDa).

### Fluorescence Titration

The 2-mL PBS solution of fluorescein (**2**) (6 μM) was titrated by successive addition of Cage **1** (2.5 μL, 2.4 mM in DMSO). The total volume change during the titration is negligible. A spectra range between 500 and 650 nm was monitored during the titration.

### Job Plots From UV-Vis Spectroscopy

A total of 11 samples containing Compound **1** and **2** in PBS were prepared in different molar ratio where each molar fraction varied from 0 to 1.0. The total concentration of **1** and **2** remained as constant as 10 μM. Solutions were prepared freshly and observed from 450 to 650 nm. Abs (506 nm) –ε (fluorescein) × [fluorescein] – ε (**1**) × [**1**] was plotted against the molar fraction of fluorescein, where Abs (506 nm) is the absorption of the sample at 506 nm, ε(fluorescein) is the extinction coefficient of fluorescein measured at 506 nm, ε(**1**) is the extinction coefficient of Cage **1** measured at 506 nm, and [**2**] and [**1**] are the concentration of fluorescein and Cage **1** within the sample.

### Dialysis Experiments

A volume of 1 mL PBS solution (pH = 7.4) or acetate buffer (pH = 5.0) containing Cage **1** ([Pt] = 120 μM) or nMOC ([Pt] = 240 μM) were sealed in micro-dialysis bags (10 kDa MWCO) against 500 mL PBS solution or acetate buffer at R.T. A series of samples were collected within the bags for every few hours and analyzed with GFAAS. All the measurements were done in triplicate.

### Cellular Uptake Evaluated by GFAAS

One million A2780cis cells were seeded on 60 × 10 mm petri dishes and incubated at 37°C overnight. These cells were treated with cisplatin ([Pt] = 25 μM), **1** ([Pt] = 25 μM), and nMOC ([Pt] = 25 μM) for 4 h at 37°C. The remaining alive cells were harvested by trypsinization and digested in 200 μL 70% HNO_3_ at R.T. overnight. The platinum content in the cells were analyzed by GFAAS. All experiments were performed in triplicate.

### Cellular Uptake Evaluated by Flow Cytometry

A2780cis cells were seeded on 60 × 10 mm petri dishes and incubated at 37°C overnight. These cells were treated with PBS, fluorescein ([**2**] = 3 μM), fluorescein ([**2**] = 3 μM) plus polymer, and nMOC ([**2**] = 3 μM) for 4 h at 37°C. The cells were harvested by trypsinization and with the Accuri flow cytometer and the cell populations were analyzed using the FlowJo software.

### Flow Cytometric Analysis

A2780cis cells were seeded on 60 × 10 mm petri dishes and incubated at 37°C overnight. A2780cis cells were treated with the Pt compounds (nMOC [Pt] = 4 μM; cisplatin [Pt] = 10 μM) at 37°C for 72 h. The samples were analyzed with the Accuri flow cytometer and the cell populations were analyzed using the FlowJo software. Annexin V early apoptosis detection kit was used for apoptosis analysis, and the experiments were carried out by following the standard protocol supplied by the manufacturer.

### Immunoblotting Analysis

A volume of 0.6 ml of RIPA buffer was added to the A2780cis cells treated with the different Pt compounds (nMOC [Pt] = 2.5, 5.0, and 7.5 μM, 48 h, and Cage **1** [Pt] = 2.5, 5.0, 7.5 μM, 48 h). The cells were gently shaken for 15 min at 4°C. Then, the cells were collected with a cell scraper, and transferred to a microcentrifuge tube. A total of 100 μg whole cell lysate with SDS loading dye was boiled for 2–3 min. A volume of 50 μl of lysate and loading dye mixture per well was loaded for gel electrophoresis (120 Volts for 90 min). Afterward, the proteins were transferred from the gel to UltraCruz® Nitrocellulose Pure Transfer Membranes, using an electroblotting apparatus according to the manufacturer's protocols. Non-specific binding was blocked using Blocker™ Blotto (30–60 min at R.T.). The blocked membrane was incubated with primary antibody with 1:1,000 dilution in Blotto for 1 h at R.T. with shaking. The membrane was washed three times for 5 min each with Tris-buffered saline, pH 7.5 with 0.5% Tween 20 (TBST). The membrane was incubated with horse-radish peroxidase (HRP) conjugated secondary antibody with dilution 1:2,000 for 1 h at R.T., with shaking. The membrane was washed three times for 5 min each with TBST. Chemiluminescence Luminol Reagent was used for detecting according to the standard protocol supplied by the manufacturer.

### MTT Assays

Cells were seeded on a 96-well plate (2,000 cells per well) in 200 μL RPMI or DMEM and incubated for 24 h at 37°C. On the following day, the cells were treated with the platinum compounds of 50 μL, separately at varying concentrations for 72 h at 37°C. The cells were then treated with 30 μL fresh medium containing 3-(4,5-dimethylthiazol-2-yl)-2,5-diphenyltetrazolium bromide (MTT) (5 mg/mL) and incubated for 4 h at 37°C. The medium was removed, 200 μL of DMSO was added to the cells, and the absorbance of the purple formazan was recorded at 565 nm using a BioTek Elx800 microplate plate reader. Each experiment was performed in triplicate for each cell line. The IC_50_ values were derived from Dose Response curves to obtain the Pt concentration that could reduce cell viability to 50%. All the IC_50_ values were obtained by fitting experimental data using Origin.

### LIVE/DEAD Cell Viability Assay

A2780cis cells were cultured on 35 mm sterile glass bottom culture dishes (MATTEK Corporation) for 24 h at 37°C. The cells were then treated with cisplatin ([Pt] = 10 μM), nMOC ([Pt] = 10 μM) for 48 h at 37°C. The LIVE/DEAD assay was carried out by following the standard protocol supplied by the manufacturer.

## Results and Discussion

### Preparation of the Pt_6_L_4_ Metal-Organic Cage (1)

The M_6_L_4_ type of metal-organic cages was first developed by Fujita (Ibukuro et al., 1998). Recently, Lippard et al. reported that a Pt_6_L_4_ cage bearing a bpy aromatic chelator is able to trigger DNA damage and apoptosis via non-covalent binding (Zheng et al., 2016). In this report, we developed a Pt_6_L_4_ Cage **1** bearing a larger aromatic chelator, 1,10-phenanthroline (phen), toward generating severe DNA damage and high potency. As shown in Figure 1A, Cage **1** is an octahedral cage formed by self-assembly of six 1,10-phenanthroline-Pt(II) centers and four 2,4,6-tris(4-pyridyl)-1,3,5-triazine ligands (L). It is synthesized by following a reported procedure using microwave synthesis, and self-assembly was confirmed by NMR spectroscopy. The proton NMR spectrum of **1** (Figure 1B) matched the previous reports and the integrations confirmed a 6:4 ratio between 1,10-phenanthroline-Pt(II) centers and the pyridyl ligands (Zheng et al., 2016).

### Cellular Responses of the Pt_6_L_4_ Metal-Organic Cage (1)

**Figure 2:**
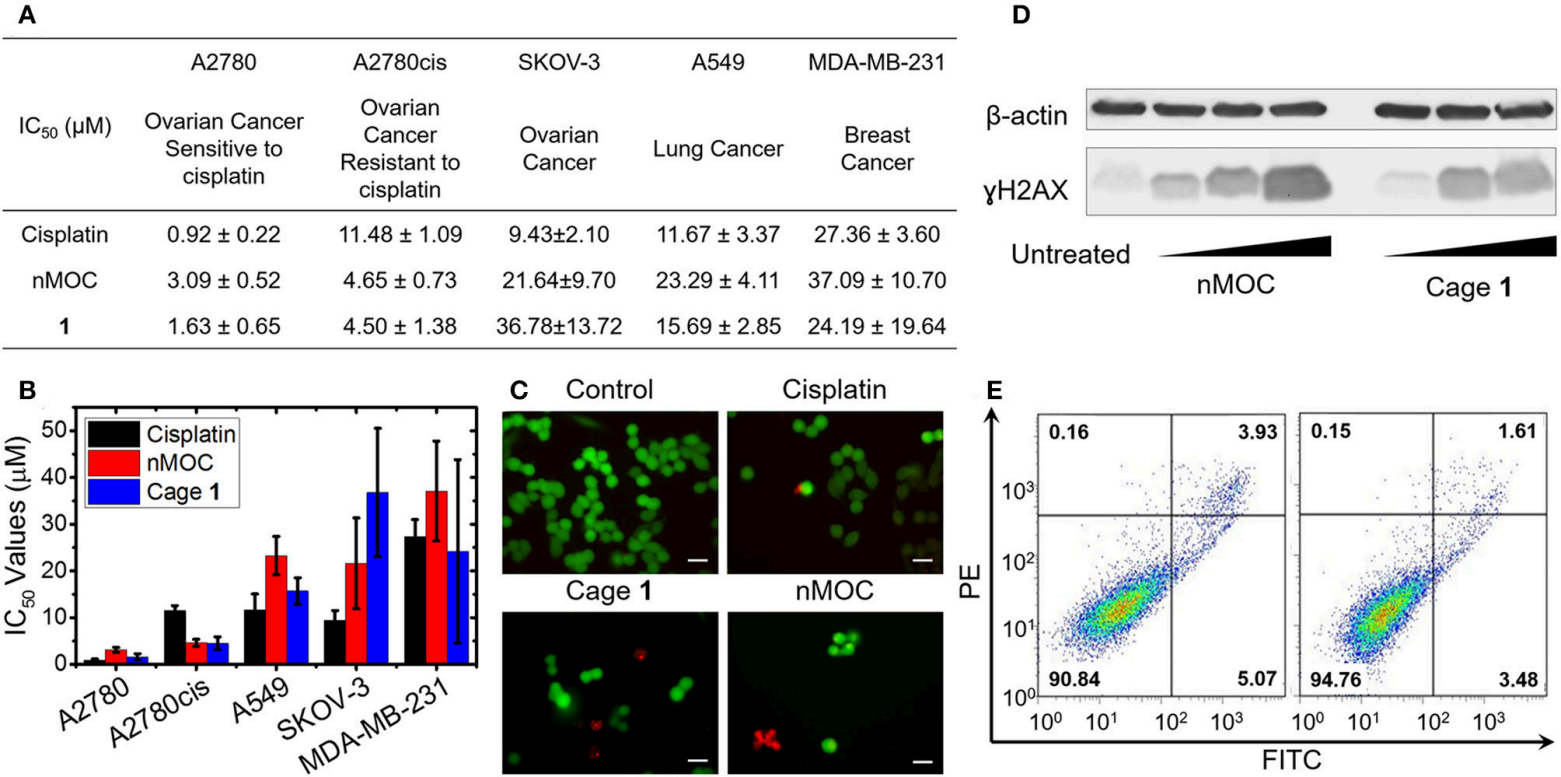
Cellular responses of the metal-organic cage **(1)** and the nanoparticles of MOCs. **(A)** A table of the IC_50_ values of cisplatin, **1**, and nMOC determined by MTT assays; **(B)** Bar graph of IC_50_ values obtained using a panel of human cancer cell lines; **(C)** Fluorescence microscopic images obtained for the live/dead cell assay using A2780cis cells treated with cisplatin, **1**, or nMOC; **(D)** Immunoblotting analysis of γH2AX in A2780cis cell line; **(E)** Annexin V apoptosis assay using A2780cis cells treated with nMOC (left) and cisplatin (right).

The cytotoxicity profiles of the metal-organic cage (**1**) was determined against a panel of human cancer cell lines using MTT [3-(4,5-dimethylthiazol-2-yl)-2,5-diphenyltetrazolium bromide] assays. As shown in Figures 2A,B, five human cancer cell lines were tested, including ovarian cancer cell line A2780, cisplatin-resistant ovarian cancer cell line A2780cis, ovarian cancer cell line SKOV-3, lung cancer cell line A549, and triple-negative breast cancer cell line MDA-MB-231. The cells were incubated with Cage **1** or cisplatin for 72 h, and the IC_50_ values (concentrations required to induce 50% viability) were derived from dose–response curves (Figure 2A). All of the IC_50_ values refer to platinum concentrations. As shown in Figures 2A,B, in A2780, A549, and MDA-MB-231, **1** exhibits IC_50_ values comparable to those of cisplatin. Notably, **1** exhibits higher cytotoxicity against A2780cis (IC_50_ = 4.50 ± 1.38 μM) than cisplatin (IC_50_ = 11.48 ± 1.09 μM). As A2780cis cells are intrinsically resistant to cisplatin, this data shows that **1** has the potential to treat chemoresistant ovarian cells. Furthermore, we used immunoblotting analysis to evaluate the DNA damage triggered by Cage **1**. As shown in Figure 2C, A2780cis cells incubated with **1** ([Pt] = 2.5, 5.0, and 7.5 μM) for 48 h showed a marked increase in phosphorylated H2AX (γH2AX), a classic biomarker for DNA damage. The results of MTT assays and immunoblotting analysis clearly support that Cage **1** is able to induce DNA damage and exhibit high potency against chemoresistant ovarian cancer cells. However, we found that this cage structure exhibits very limited solubility in cell culture media (<20 μM), due to the high hydrophobicity of the large aromatic components. Therefore, additional formulation is essential to promote the solubility of this hydrophobic cytotoxic cage.

### Nanoformulation of the Metal-Organic Cage (1) Into nMOC

**Figure 3:**
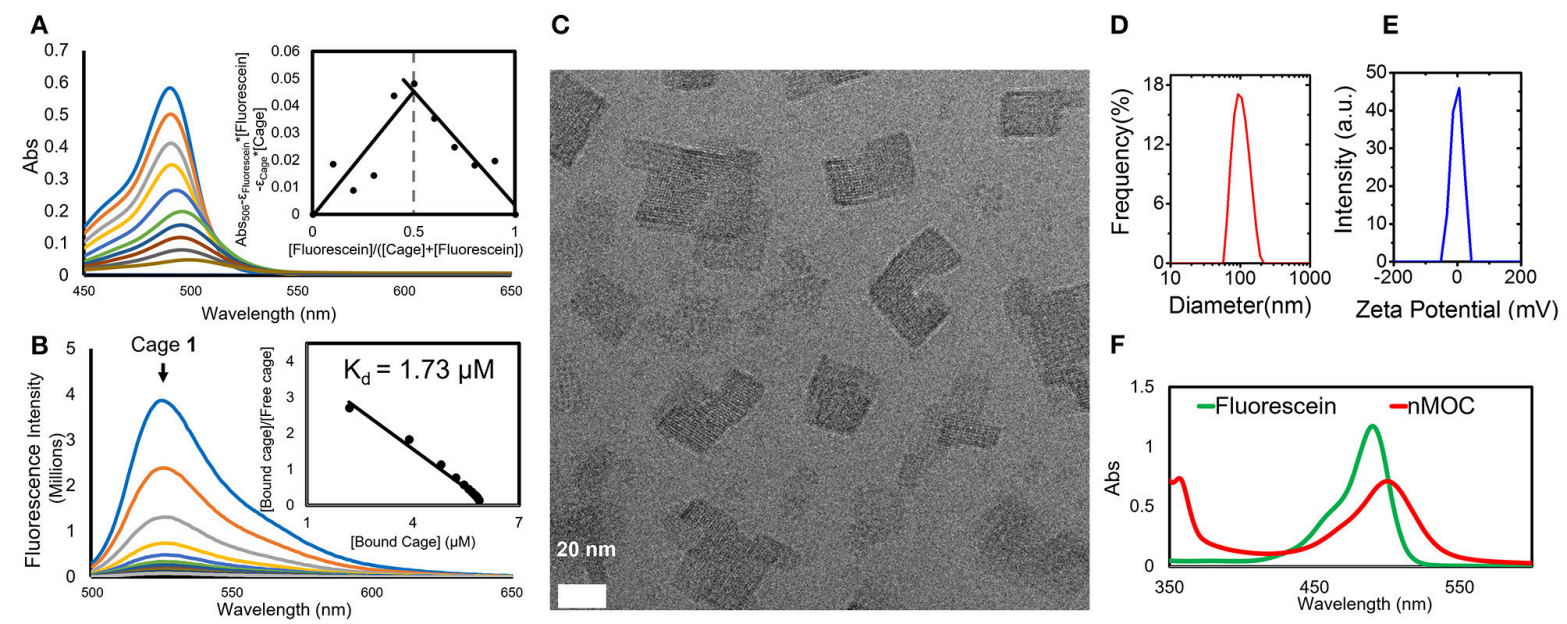
Nanoformulation of Cage **1** using fluorescein **(2)** and MPEG_5k_-PGA_50_ **(3)**. **(A)** Quenching of fluorescence emission of fluorescein by titration with **1** (6 μM) and the Scatchard plot extracted from fluorescence titration to determine K_d_; **(B)** Changes of the absorption spectra of the mixture of **1** and **2** in a different molar ratio (0–1.0) and the Job plot extracted from titration to determine the binding ratio of **1** and **2**.; **(C)** A cryo-EM image (Scale bar: 20 nm) **(D)** DLS analysis of size distribution; **(E)** Zeta-potential analysis; **(F)** UV-vis spectra of fluorescein and nMOC in PBS ([**2**] = 20 μM).

We found that combination of Cage **1** with fluorescein (**2**) and MPEG_5k_-PGA_50_ (**3**) can produce nanoparticles of the metal-organic cages (nMOC) in aqueous solution. In the formulation experiment, the DMSO solution containing the metal-organic cage (**1**) (90.0 μL, 2.00 mM) was added to 1 mL PBS solution of MPEG_5k_-PGA_50_ (**3**) (1.00 mg/mL) and fluorescein (**2**) (30.0 μL, 13.0 mM) with shaking at R.T. The nanoparticles were purified via centrifugal filtration, and the yield of the nanoformulation is 56.7%. As determined by GFAAS, upon nanoformulation, the concentration of **1** can reach up to 0.4 mM in aqueous solution and cell culture media. This nanoformulation is driven by the host-guest interactions and electrostatic interactions between the three components. During the formulation, significant color change from yellow green to red was observed, which is associated with the formation of the host-guest complex between Cage **1** and fluorescein (**2**). UV-vis and fluorescence spectroscopy were applied to understand such host-guest interactions. The chromatic change was characterized by UV-vis spectroscopy shown in Figure 3A. Increasing the molar ratio of **1** leads to red shift of the absorption spectra of **2**. Using a Job plot extracted from the UV-vis studies (the insert of Figure 3A), we determined the binding ratio of the cage and fluorescein to be 1:1. To further measure the dissociation constant (K_d_) of the host-guest complex, we performed fluorescence titration of Cage **1** to the fluorescein (**2**) solution. As shown in Figure 3B, gradual addition of Cage **1** into the PBS solution of fluorescein (6 μM) resulted in the decrease in the fluorescence intensity at 525 nm. As extracted from a Scatchard plot (the insert of Figure 3B), the experimental K_d_ value was determined as 1.73 μM. These results are consistent with our previous report using a similar cage structure, and it is believed that the fluorescein molecule is encapsulated within the cavity of Cage **1** (Yue et al., 2018a). As the final product of the formulation, nMOC encapsulating fluorescein was obtained in the presence of the pegylated anionic polymer (**3**). Driven by the electrostatic interactions, the cationic host-guest complex binds to the anionic polymer, leading their aggregation in aqueous solution. By virtue of the PEG group of the polymer, such aggregations were stabilized on a nanoscale. In the cryo-EM image (Figure 3C), the nanoaggregation of the cages was clearly observed, and they are around 20–40 nm. These nanocores are stabilized by their peripheral PEG. As measured by DLS (Figure 3D), the average size of the overall nanoparticles is 98.0 ± 8.2 nm. The particle size difference between TEM and DLS is likely due to the peripheral PEG. The PEG layer of the nanoparticles cannot be detected under TEM. In addition, due to peripheral PEG, the zeta potential of the nanoparticles is close to zero (−1.0 ± 0.4 mV) (Figure 3E). The integrity of the cage structure within the nanoparticles was supported by the UV-vis studies. In the UV-vis spectra (Figure 3F), the peak corresponding to nMOC is red-shifted compared to that of free fluorescein, and this is due to the encapsulation of fluorescein by Cage **1** within the nanoparticles. The combined evidence from spectroscopic analysis, GFAAS, TEM, and DLS clearly support that the hydrophobic cage can be readily formulated in well-defined nanoparticles and increase its solubility in aqueous solution.

### Cellular Uptake and Drug Release of nMOC

**Figure 4:**
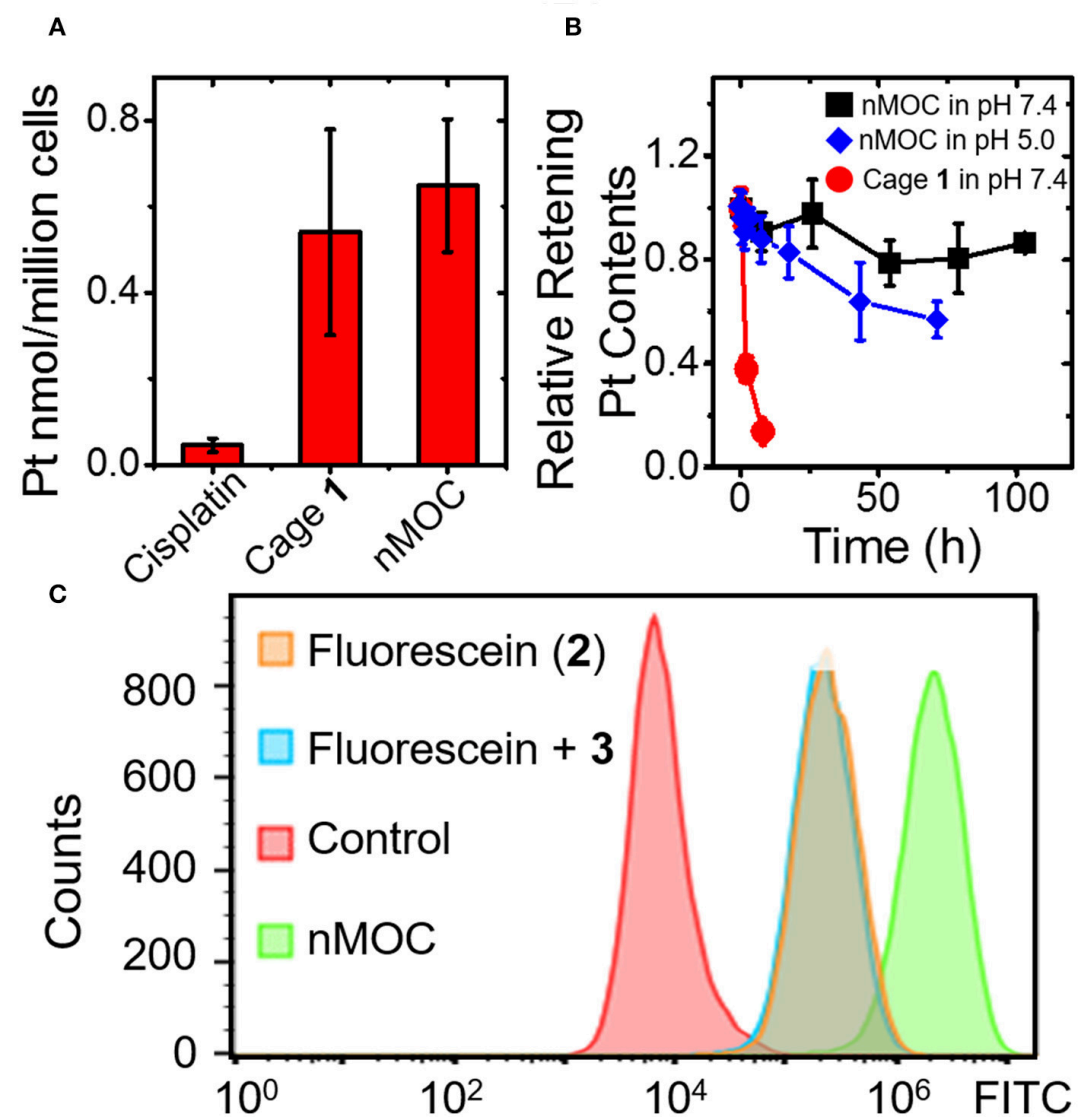
Cellular uptake drug release profiles of the nanoparticles of MOCs. **(A)** Cellular uptake profiles of the different Pt compounds in A2780cis ovarian cancer cells analyzed by using GFAAS; **(B)** Drug release profile of nMOC and Cage **1** in PBS (pH = 7.4) or acetate buffer (pH = 5.0) at R.T.; **(C)** Cellular uptake profiles analyzed by using flow cytometry.

Next, we examined whether the nMOC can enter cancer cells and release Cage **1** and fluorescein (**2**). We determined the cellular uptake of the nanoparticles and compared that with cisplatin and MOCs using GFAAS. The results (Figure 4A) show that the cellular uptake of cisplatin was only 50 pmol/million cells in A2780cis cells, while the uptakes of MOCs and Cage **1** were 10 times higher. This results verified that the nanoparticles can readily enter cancer cells. Furthermore, the drug release profiles of these nanoparticles under different pH were analyzed using dialysis in micro-dialysis bags (10 kDa MWCO). In Figure 4B, the nanoparticles show a very slow release in PBS (pH = 7.4), and there's only 20% Pt contents released after 100 h. As a control, Cage **1** exhibits fast escape from the dialysis bag under the similar condition, the half-life of which is only less than 2 h. In acidic acetate buffer (pH = 5.0), the nanoparticles rapidly release the Pt contents. As shown in Figure 4B, almost half of the Pt contents were released within 72 h in the acetate buffer. Therefore, these nanoparticles are expected to stably hold the therapeutic contents before entering cells and then readily release the cage in the acidic environment of endosomes. Finally, we employed flow cytometry to verify the intracellular drug release. The A2780cis cells were treated with PBS, fluorescein (**2**), **2** + **3**, and nMOC. As a result shown in Figure 4C, the sample treated **2** exhibits a relatively low fluorescence intensity, and this is because fluorescein (**2**) itself has very poor cell penetration. As a control, addition of the anionic polymer did not increase the uptake. Notably, the cells treated with the nanoparticles showed marked increase in fluorescent intensity. Considering that the fluorescence signals of **2** is quenched by Cage **1** within the nanoparticles, the intense fluorescence observed in the flow cytometric analysis is resulted from the intracellularly released fluorescein. Therefore, we conclude that nMOC act as a promising system for delivering the hydrophobic cytotoxic metal-organic cages and the encapsulated fluorescein guest.

### Cellular Responses of nMOC

Finally, the *in vitro* efficacy and cellular responses of the nanoparticles were examined. For A2780, A2780cis, A549, SKOV-3, and MDA-MB-231 cell lines in Figures 2A,B, the nanoparticles exhibit IC_50_ values comparable to those of Cage **1**. It is notable that nanoparticles maintain a lower IC_50_ value against A2780cis cells (IC_50_ = 4.65 ± 0.73 μM) compared to cisplatin (IC_50_ = 11.48 ± 1.09 μM). Resistance factors calculated based on IC_50_(A2780cis)/IC_50_(A2780) represent an important factor to evaluate how chemoresistant ovarian cancer cells response to the therapeutic candidates. As a result of the MTT assays, the nanoformulation has a much lower resistance factor compared to that of Cage **1** or cisplatin. For nMOC, the value of IC_50_(A2780cis)/IC_50_(A2780) is 1.50, while that of Cage **1** is 2.76 and cisplatin is 12.5. Furthermore, Live/Dead cell assay was used to confirm the anticancer activity of nMOC against A2780cis cells (Figure 2C). A2780cis cells treated with the nanoparticles contain a higher population of dead cells, but those treated with cisplatin remained viable. In addition, the results of the immunoblotting analysis (Figure 2D) show that the nanoparticles act as a DNA damaging agent like Cage **1** and induced phosphorylation of H2AX (γH2AX) in the treated A2780cis cells. Finally, annexin V flow cytometric analysis was used to further verify the apoptosis that is triggered by nanoparticles. Apoptosis represents one major cellular response to DNA damage. In healthy cells, phosphatidylserine (PS) is located on the cytoplasmic surface of the cell membrane, but in cells undergoing apoptosis, PS is translocated to the exterior of the plasma membrane, exposing PS to the external cellular environment where it can be detected by annexin V conjugates. By using annexin V and PI, both early and late stage apoptosis can be identified. As shown in Figure 2E, the nanoparticles induced significant populations of cells to under early- and late-stage apoptosis (5.07 and 3.93%, respectively). Variation of IC_50_ values (Figure 2A) of the platinum compounds across different human cell lines is attributed to different aspects of the mechanism of action. Different cell lines have different capabilities of exporting platinum compounds via efflux, different concentrations of intracellular glutathione that can deactivate platinum compounds, different nuclear DNA damaging repair capability, and different expression levels of anti-apoptotic proteins. Collectively, the cell-based studies suggest that the nanoformulation of Cage **1** maintains its mechanism of action of inducing DNA damage and apoptosis and exhibits high potency against chemoresistant ovarian cancer.

## Conclusion

In conclusion, we have presented a novel design of formulating cytotoxic hydrophobic metal-organic cages to facilitate their use in treating chemoresistant ovarian cancer. In this work, we demonstrated that Cage **1**, a cytotoxic hydrophobic metal-organic cage with low solubility, can be formulated into pegylated nanoparticles in presence of fluorescein (**2**) and MPEG_5k_-PGA_50_ (**3**). The nanoformulation is driven by the host-guest interactions between Cage **1** and Compound **2**, confirmed by using UV-vis and fluorescence spectroscopy. In presence of the anionic polymer (**3**), such cationic host-guest complexes precipitate in aqueous solution to form the nMOC covered by PEG, characterized by TEM and DLS. In the form of nMOC, the concentration of Cage **1** can reach up to 0.4 mM in aqueous solution or cell culture media. Upon entering cancer cells, the nMOC can slowly releases therapeutic content (**1**) and the fluorophore (**2**). Using MTT assays, we determined that the nMOC exhibits promising *in vitro* efficacy against a panel of human cancer cell lines, and notably, the nMOC shows a much lower resistance factor against chemoresistant ovarian cancer cell lines comparing to cisplatin. We envision that this study can deliver a threefold impact to the research field that focuses on applying metallosupramolecular coordination complexes in cancer therapy: First, this work demonstrates the feasibility of using pegylated anionic polymers to formulate hydrophobic cationic metal-organic cages toward promoting their solubility and biocompatibility, and ideally, this strategy can be applied to other metal-organic complexes. Moreover, this strategy also introduces the host-guest chemistry that can be further exploited for the delivery of different therapeutic entities, and this may further enhance the therapeutic effects of such systems. Finally, the promising therapeutic effects of the resulted nMOC make them possible therapeutic candidates for future studies toward treating chemoresistant ovarian cancer.

## Author Contributions

HW and ZY: synthesis and characterization of Cage **1**; HW and HD: spectroscopic studies of the host-guest complex formed by Cage **1** and fluorescein; HW and AJ: preparation and characterization of nMOC; ZQ: cell-based studies; HL and BD: analysis of phosphorylation of H2AX by western blots; Y-RZ: develop the concept and design the experiments; Y-RZ and DB: prepare the manuscript.

### Conflict of Interest Statement

The authors declare that the research was conducted in the absence of any commercial or financial relationships that could be construed as a potential conflict of interest.
